# Machine learning for microwave optimization using simplex surrogates, dual-resolution computational models and local tuning with sparse sensitivity updates

**DOI:** 10.1038/s41598-025-28208-x

**Published:** 2025-11-17

**Authors:** Slawomir Koziel, Anna Pietrenko-Dabrowska

**Affiliations:** 1 Engineering Optimization & Modeling Center, Reykjavik University, Reykjavik, 102 Iceland; 2 Faculty of Electronics, Telecommunications and Informatics, Gdansk University of Technology, Gdansk, 80-233 Poland

**Keywords:** Microwave design optimization, Computer-aided design, Global search, Machine learning, Surrogate modeling, Principal directions, Engineering, Electrical and electronic engineering

## Abstract

Numerical optimization procedures are now an integral part of the microwave design process. Ensuring reliability requires conducting parameter tuning at the electromagnetic (EM) analysis level. This, however, entails considerable computational costs. Additionally, global optimization is often necessary (e.g., multimodal problems, large-scale operating frequency re-design, design of metasurfaces), which is incomparably more expensive when using conventional techniques. In this work, we introduce a novel approach to the fast globalized optimization of microwave structures. Our methodology is founded on processing the operating parameters of the circuit rather than its complete frequency characteristics, and the utilization of simplex-based regressors. Both permit regularizing the objective function, which facilitates and speeds up the identification of the optimum design. Further acceleration is enabled by employing dual-fidelity EM simulations and restricted sensitivity updates at the final parameter tuning stage. The introduced algorithm has been comprehensively demonstrated using several microstrip components and proved to be superior over several benchmark approaches. Apart from reliability, its attractive features include remarkable computational efficiency (the average optimization cost corresponding to fewer than fifty EM simulations of the circuit), as well as simple implementation and handling with a small number of control parameters that do not have to be tuned to a specific problem at hand.

## Introduction

Contemporary microwave passive components are designed to satisfy stringent performance and functionality requirements (reconfigurability, multi-band operation, limited physical size, harmonic suppression^1–8^. This makes their development process a difficult undertaking. For example, the design of compact circuits often involves meandering of transmission lines or the employment of slow-wave effect (e.g^9,10^.,, which increases the geometrical intricacy of the layout and the number of adjustable parameters^11–13^. Further, accounting for losses, cross-coupling effects, or the presence of connectors necessitates the utilization of electromagnetic (EM) analysis for reliable electrical characteristic evaluation. In particular, the tuning of circuit geometry parameters must be based on EM simulations. The latter is increasingly often realized using formal optimization methods, as they permit us to handle multiple variables and design goals simultaneously. Nevertheless, the computational efficacy of EM-driven optimization, even in the local case, remains far from satisfactory. The costs are significantly higher for global search, which is, however, recommended in many situations (e.g., optimization of metamaterials/metasurfaces^14,15^, radiation pattern synthesis^16^, multi-objective design^17^, simulation-based size reduction^18^, or operating frequency relocation^19^.

Nowadays, population-based metaheuristics^20–25^ prevail in the global search. There are numerous methods within this class. Some of the more traditional but still widely used are evolutionary optimization techniques^26^, particle swarm optimization algorithm (PSO)^27^, or differential evolution^28^. Notable examples of more recent techniques are the grey wolf optimization, firefly algorithm, and harmony search (e.g^29^., -^30^. The new routines are continuously piling up^31–37^. What enables the global search potential is the exchange of data between the candidate solutions processed by the algorithm^38–40^ as well as partial randomization (e.g., local alterations referred to as mutation^41^, design relocation biased towards the current best solution^42,43^, often imitating biological or social processes. The implementation of the aforementioned methods is relatively simple. However, they are costly in computational terms. Typically, the numbers of objective function evaluations are measured in thousands per algorithm run. Consequently, EM-based optimization using such methods is generally prohibitive, and their applications are limited to handling cheap models (e.g., analytical factor models in the case of antenna arrays^44^.

Alleviating the cost-related difficulties is possible using surrogate modeling methods (kriging^45^, artificial neural networks^46^, Gaussian process regression^47^. In surrogate-assisted frameworks, data-driven models act as fast predictors replacing expensive EM analysis^48–52^ and are gradually refined by incorporating EM simulation results acquired throughout the optimization process^53^. These schemes are frequently categorized as machine learning (ML) procedures^54,55^. Modern ML frameworks often exploit Bayesian optimization (BO)^56–58^, often in conjunction with population-based metaheuristics^59^, and multi-fidelity approaches^60^, as well as reinforcement learning^61,62^ (also in combination with deep neural networks^63,64^. BO has been applied to solve various design tasks such as global optimization, uncertainty quantification, and inverse design^65,66^, but also parameter determination of neural network models of high-frequency structures^67^. Other examples of applying ML in microwave design optimization can be found in^68–74^. Casting the numerical operations onto surrogates results in a considerable computational speedup. The bottleneck is the construction of accurate data-driven models, which is thwarted by dimensionality-related issues, large domains (extensive ranges of geometry parameters), and nonlinearity of frequency characteristics. As a result, most ML algorithms are showcased using rather simplistic examples^75,76^. Some of the mentioned issues can be mitigated using domain confinement (also known as performance-driven modeling)^77–80^. The surrogate is rendered only in a small region containing high-quality designs identified beforehand^77^, reducing the necessary training dataset size. Another possibility is employing the response feature approach^81^, i.e., expressing the design task with regard to so-called characteristic points of the circuit responses. This smoothens the objective function, expediting optimization procedures^82^ or facilitating the surrogate model setup^83^.

This paper aims to introduce a novel methodology for the rapid globalized EM-driven optimization of passive microwave circuits. The proposed technique targets operating parameters of the considered circuit (e.g., center frequencies, power split ratios), which are inferred from the EM simulation data and predicted using structurally simple regression models. The latter dramatically improves the reliability of optimum identification and reduces the costs. Additional acceleration factors include the employment of dual-fidelity simulations and restricted sensitivity updates applied at the final (gradient-based) parameter tuning stage. The presented methodology has been verified with the use of several microstrip circuits optimized for different scenarios and compared to several benchmark methods: nature-inspired algorithms, random-start local search, and two machine-learning-based strategies. The results obtained corroborate our technique’s exceptional cost effectiveness (the average cost equals merely around 45 EM analyses of the structure at hand), global search potential, and the competitive quality of the designs produced. Excellent performance, supplemented by straightforward setup and handling, makes it a practical alternative to existing globalized search techniques for EM-driven microwave design.

This paper brings together several original components and technical contributions, which can be summarized as follows: (i) the development of a novel framework for global optimization of microwave components, (ii) the incorporation of algorithmic tools oriented towards low-cost global exploration of the parameter space such as low-resolution parameter pre-screening, feature-based simplex surrogates, and global search stage through simplex evolution, (iii) rapid final tuning involving variable-fidelity EM simulations and sparse sensitivity updating strategies based on principal directions, (iv) comprehensive demonstration of the algorithm’s efficacy and superiority over a range of bio-inspired, gradient-based, and machine learning methods. The main novelty of the work lies in meticulous integration of several mechanisms (as mentioned above) that work in synergy and contribute to both computational efficiency and reliability.

## Globalized microwave optimization by operating parameter handling, dual-fidelity em simulations, and restricted sensitivity updates

In this section, we elaborate on the details of the introduced optimization methodology. The design task definition (Sect. 2.1), and comments on dual-fidelity EM models (Sect. 2.2), are followed by the definition of simplex-based surrogates in Sect. 2.3. The global and local search parts of the entire framework are discussed in Sect. 2.4 and 2.5, whereas Sect. 2.6 outlines the complete procedure.

### EM-Driven optimization task definition

**Fig. 1:**
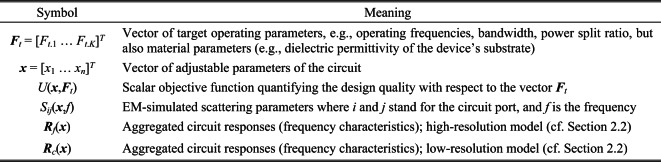
Notation used in the context of EM-driven microwave design formulation.

The notation relevant to simulation-based optimization of microwave components can be found in Fig. 1. The fundamental role is played by the scalar merit function *U*(***x***,***F***_*t*_), which is defined with respect to the adjustable parameter vector ***x***, and the target operating parameter vector ***F***_*t*_. The constraints or multiple objectives are treated using the penalty function approach^84^. The specific formulations of the objective functions employed in our work are listed in Sect. 3. It should be noted that other ways of handling multiple design goals are available as well, e.g., the weighted sum approach^85^ or genuine multi-objective optimization^86–88^.

With the notation introduced in Fig. 1, the microwave optimization problem can be stated as a nonlinear minimization task

1 $${{\bf{x}}^*} = \arg \mathop {\min }\limits_{\bf{x}} U({\bf{x}},{{\bf{F}}_t})$$

In (1), x^*^ is the optimum parameter vector to be found.

### Dual-Fidelity EM simulations

Simplified representations, both analytical ones and equivalent network models, have been the primary tools of microwave design for decades^89^. Yet, the complexity of contemporary passive circuits makes such representations insufficient, leaving full-wave EM models the only means to capture cross-coupling effects, losses, dielectric anisotropy, etc. By controlling the discretization level of the considered (and other changes in the model^90^, one can alter the trade-offs between the evaluation speed and reliability, which is a convenient way of rendering less accurate but faster models. Due to sharing the same underlying physics, the simulation results of various fidelities are typically well-correlated, allowing for using lower-resolution models as reliable predictors upon suitable correction^91,92^. Normally, two resolution levels are utilized in practice^93^, although model management strategies involving a continuous spectrum of fidelities are also employed^94^.

We employ two models: low- and high-resolution ones, ***R***_*c*_(***x***) and ***R***_*f*_(***x***), respectively. To boost the computational efficiency of our algorithm, the model ***R***_*c*_ will be used for sampling (pre-screening) and performing the global search stage of the optimization process. The simplex-based regression models in this stage will also be constructed using ***R***_*c*_. The model ***R***_*f*_ will only be used for the final tuning of the circuit parameters (the last stage of the process), which is necessary to ensure reliability.

### Surrogate modeling of circuit operating parameters

Global optimization is a computationally expensive endeavor, which can be facilitated using surrogate modeling methods^95–98^. The primary issue is the construction of reliable metamodels of highly nonlinear outputs of microwave components over large parameter spaces. This section discusses the proposed approach to this problem. It capitalizes on changing the focus of the modeling process from frequency responses to the system’s operating parameters.

#### Frequency responses versus operating parameters

**Fig. 2:**
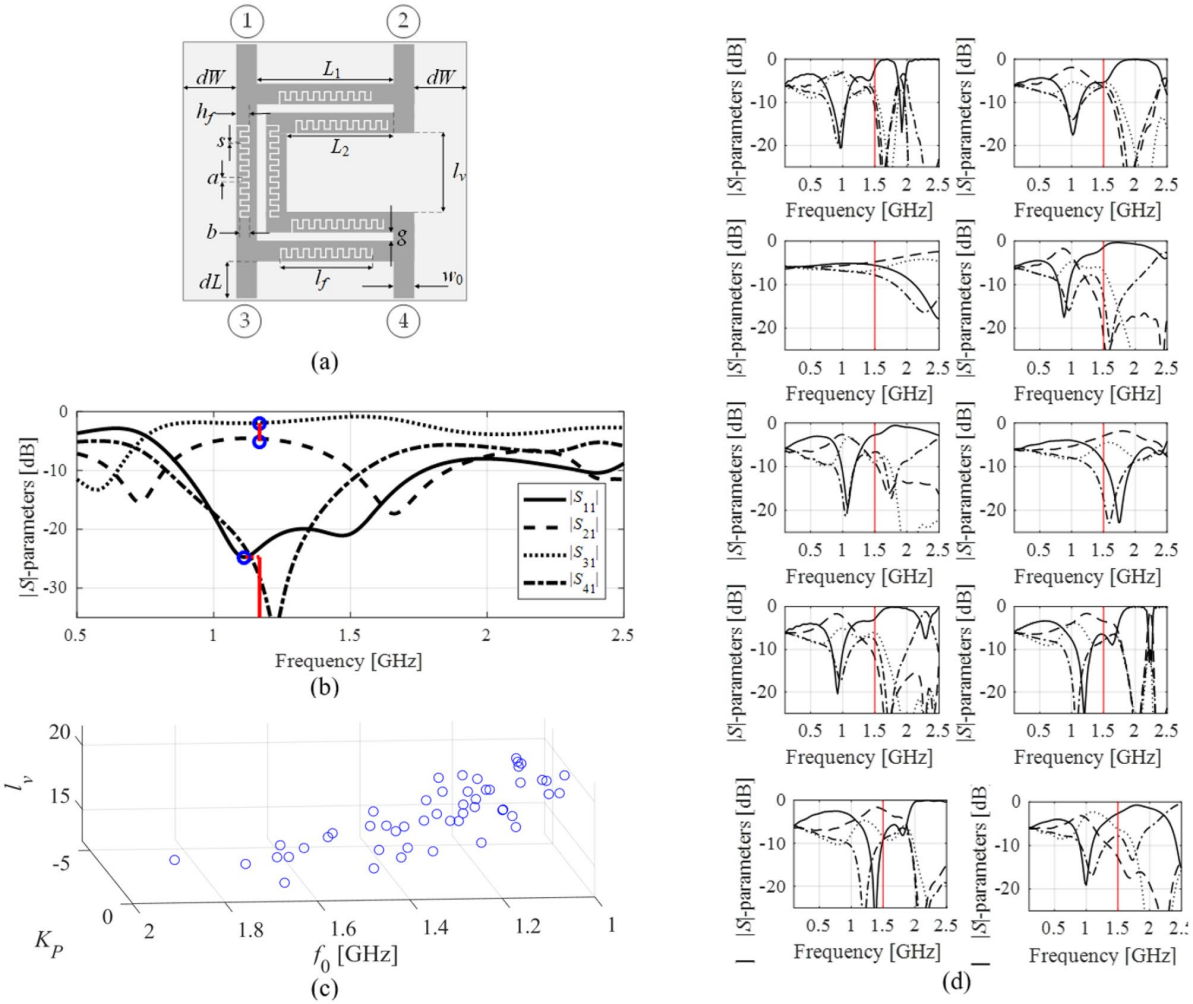
*S*-parameter characteristics of a rat-race coupler: (a) circuit layout, (b) approximated operating parameters of the coupler: the power division is assessed for the frequency calculated as the mean value of the |*S*_11_| and |*S*_41_| minima (marked using thick vertical line), which is also used as the approximation of the circuit’s operating frequency *f*_0_; (c) relations between the operating parameters and selected circuit dimensions. The plots are created using a set of randomly generated designs. Clear patterns are visible even though the trial points were not optimized whatsoever; (d) *S*-parameters simulated for parameter vectors randomly allocated across the design space considered. The intended operating frequency of the circuit is marked with vertical line. Local optimization fails when starting from most of the designs shown: the misalignment between the target and the actual operating conditions is too severe.

As mentioned earlier, modeling of nonlinear characteristics of microwave components across extended ranges of frequency and geometry parameters is a daunting task. For example, consider a rat-race coupler of Fig. 2(a) and its EM-simulated *S*-parameters (Fig. 2(b)). Clearly, a successful optimization of the circuit through local search (here, for a target frequency of *f*_0_ = 1.5 GHz) is contingent upon the initial design quality. For most of the points shown in Fig. 2(d), local optimization would fail. Moreover, constructing a reliable surrogate model of circuit responses seems to be extremely difficult.

However, the situation observed from the angle of the operating parameters renders an entirely different picture. As shown in Fig. 2(c), the geometry parameters are interrelated with the operating figures (here, the center frequency *f*_0_ and the power division ratio *K*_*P*_) in a considerably simpler manner. The said dependence can be described as monotonic, even though the plots have been generated using random samples (without any prior optimization). This type of relationship is common for high-frequency circuits, as proven both for optimization and modeling^81,82^-^83,99,100^.

#### Regression modeling using simplex-based design of experiments

We aim to explore the relationships illustrated in Fig. 2 for reduced-cost globalized optimization of microwave components. The simplicity of these dependencies fosters the utilization of structurally simple surrogate models to represent the operating conditions of the circuit. Here, we choose a simplex as a suitable geometrical object, which is spanned by *n* + 1 affinely independent vectors playing a role in the training dataset (with the parameter space dimensionality denoted as *n*). Below, we define the simplex-anchored surrogates and explain their employment in optimization. We refer to the notation gathered in Fig. 4. Therein, the fundamental concepts include the operating vectors ***f*** and performance vectors ***l***. Let us explain these quantities using an exemplary microwave coupler. The frequencies *f*_1_ and *f*_2_ of the minima of |*S*_11_| and |*S*_41_| characteristics, respectively, are collected in the operating figure vector. While the levels of the said responses (i.e., *l*_1_ and *l*_2_) along with the power division at the frequency (*f*_1_ + *f*_2_)/2 are assembled in the performance figure vector. Thus, we have, ***f*** = [*f*_1_ *f*_2_]^*T*^ and ***l*** = [*l*_1_ *l*_2_ *l*_3_]^*T*^.

**Fig. 3:**
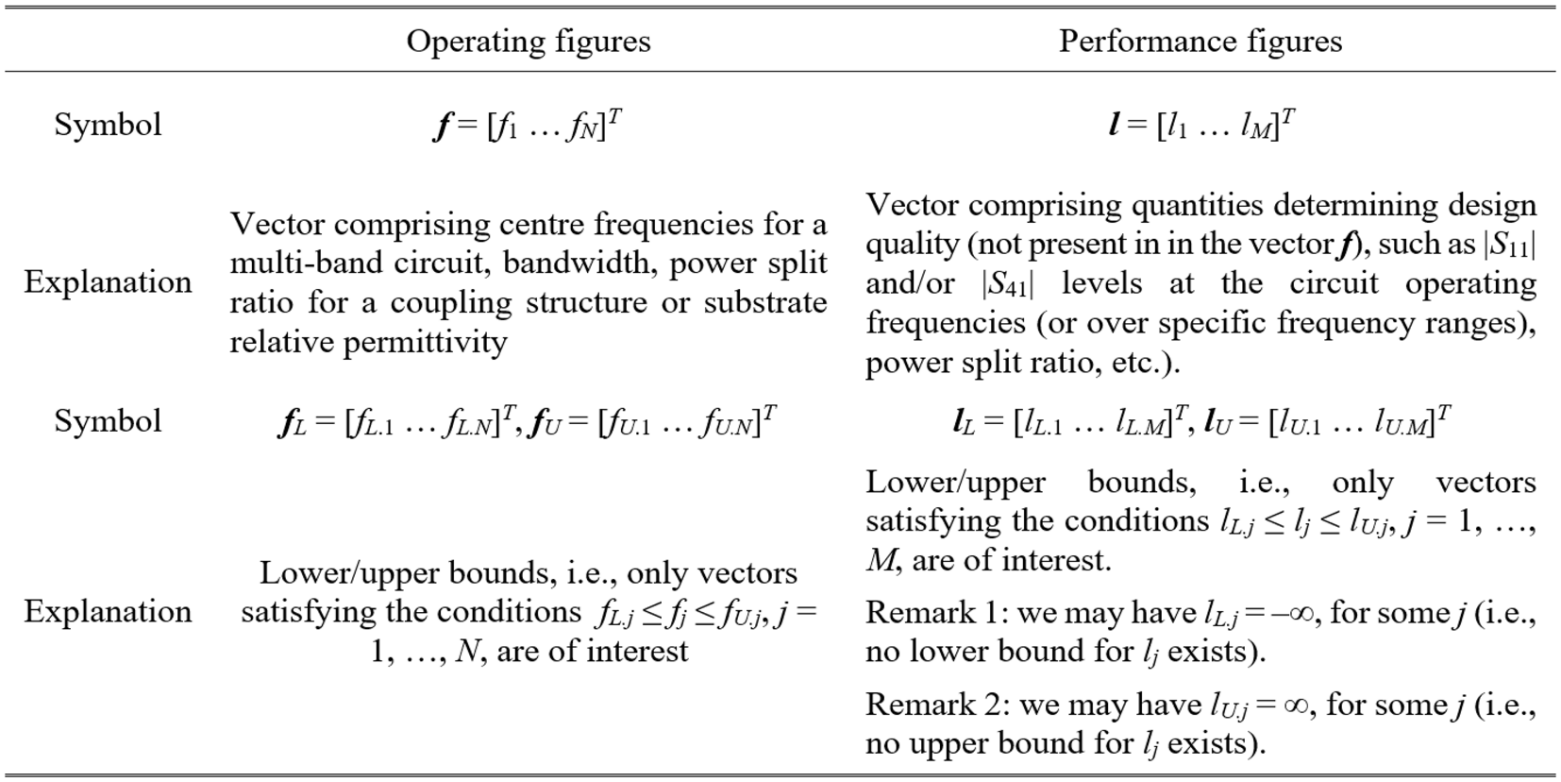
Simplex-based surrogate models: notation and terminology.

Consider *n* + 1 affinely independent ***x***^(*j*)^ = [*x*_1_^(*j*)^ … *x*_*n*_^(*j*)^]^*T*^, *j* = 0, …, *n*, in the design space *X* (which is bounded by parameters’ lower and upper limits, i.e., circuit dimensions). The corresponding operating and performance vectors are ***f***^(*j*)^ = ***f***(***x***^(*j*)^) = [*f*_1_^(*j*)^ … *f*_*N*_^(*j*)^]^*T*^ and ***l***^(*j*)^ = ***l***(***x***^(*j*)^) = [*l*_1_^(*j*)^ … *l*_*M*_^(*j*)^]^*T*^, respectively. In practical implementation, the vectors ***x***^(*j*)^ are randomly acquired until the required number of points satisfying the conditions.

provided in Fig. 3 have been identified. In other words, only the designs satisfying the said conditions form the basis for simplex-based regression model construction.

#### Surrogate model definition

Consider the affinely independent set {***x***^(*j*)^} introduced in Sect. 2.3.2. Since the vectors ***x***^(*j*)^ – ***x***^(0)^ are linearly independent, uniqueness of the expansion is ensured

where ***x*** is an arbitrary vector in the parameter space *X*, and

3 $${\bf{X}} = \left[ {{{\bf{x}}^{(1)}} - {{\bf{x}}^{(0)}}\;\; \cdots \;\;\;{{\bf{x}}^{(n)}} - {{\bf{x}}^{(0)}}} \right]$$

***X*** being an invertible *n* × *n* matrix. The coefficients ***a*** = [*a*_1_ … *a*_*n*_]^*T*^ are identified as

4 $${\bf{a}}({\bf{x}}) = {{\bf{X}}^{ - 1}}({\bf{x}} - {{\bf{x}}^{(0)}})$$

We aim at defining the following surrogate models: (i) ***F***(***x***) : *X* → *F*, which represents the operating parameters of the circuit, and (ii) ***L***(***x***) : *X* → *R*^*M*^, predicting its performance parameters. The definitions involve the expansion (3), (4) as well as the vectors ***f***(***x***^(*j*)^) and ***l***(***x***^(*j*)^) associated with the anchor points ***x***^(*j*)^. We have

6 $$\eqalign{ & {\bf{L}}({\bf{x}}) = {{\bf{l}}^{(0)}} + \sum\limits_{j = 1}^n {{a_j}} ({{\bf{l}}^{(j)}} - {{\bf{l}}^{(0)}}) = {{\bf{l}}^{(0)}} + {{\bf{X}}_l}{\bf{a}}({\bf{x}}) \cr & = {{\bf{l}}^{(0)}} + {{\bf{X}}_l}{{\bf{X}}^{ - 1}}({\bf{x}} - {{\bf{x}}^{(0)}}) \cr}$$

in which

7 $${{\bf{X}}_f} = \left[ {{{\bf{f}}^{(1)}} - {{\bf{f}}^{(0)}}\;\;\; \cdots \;\;\;{{\bf{f}}^{(n)}} - {{\bf{f}}^{(0)}}} \right]$$

8 $${{\bf{X}}_l} = \left[ {{{\bf{l}}^{(1)}} - {{\bf{l}}^{(0)}}\;\;\; \cdots \;\;\;{{\bf{l}}^{(n)}} - {{\bf{l}}^{(0)}}} \right]$$

Note that the models (5) and (6) interpolate the basis points, in particular, ***F***(***x***^(*j*)^) = ***f***^(*j*)^ and ***L***(***x***^(*j*)^) = ***l***^(*j*)^ for *j* = 0, …, *n*.

**Fig. 4:**
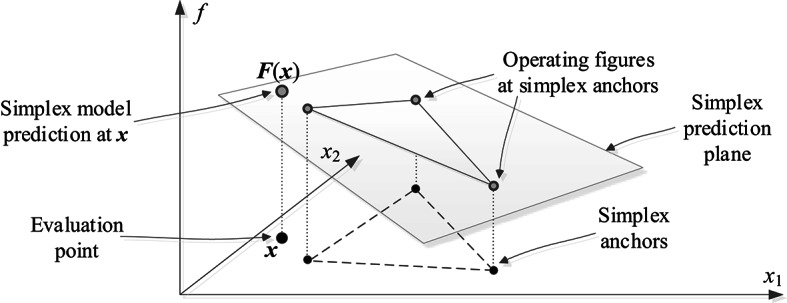
provides an illustration of simplex model construction and its use for predicting the circuit operating figures.

Figure 4. A graphical illustration of the simplex model construction and its application to predict the circuit’s operating figures ***F***(***x***) at the evaluation point ***x***.

### ML-Enabled global search

The global optimization part is executed using the surrogates ***F***(***x***) and ***L***(***x***) of Sect. 2.3.3. The underlying advantage is the aforementioned mildly nonlinear relationship of the operating parameters of the circuit with its geometrical dimensions, which “flattens” the objective function profile, making the identification of the optimal design faster and easier. The search process follows the machine learning principles, where the candidate design generated by optimizing the surrogates is employed to refine both ***F***(***x***) and ***L***(***x***). The criterion for infill selection is driven by the anticipated enhancement of the objective function.

#### Design assessment

The design quality ***x*** is assessed using the objective function *U*_*F*_ being a function of the vectors ***f***(***x***) and ***l***(***x***) (i.e., operating and performance vectors). The goal is to align ***f***(***x***) with the target ***f***_*t*_ as closely as possible. The latter is not the same as the target vector ***F***_*t*_. To explain this, consider the coupler of Fig. 2. If the device should operate at the center frequency *f*_0_ and yield a power split radio *K*_*P*_, we have ***F***_*t*_ *=* [*f*_0_ *K*_*P*_]^*T*^. However, the operating parameter vector would be defined as ***f*** = [*f*_1_ *f*_2_]^*T*^. Consequently, for consistency, one needs to take ***f***_*t*_ = [*f*_0_ *f*_0_]^*T*^.

A formal definition of *U*_*F*_ is as follows.

9 $${U_F}({\bf{x}}) = U({\bf{f}}({\bf{x}}),{\bf{l}}({\bf{x}})) = {U_L}({\bf{l}}({\bf{x}})) + {\beta _F}||{\bf{f}}({\bf{x}}) - {{\bf{f}}_t}|{|^2}$$

**Fig. 5:**
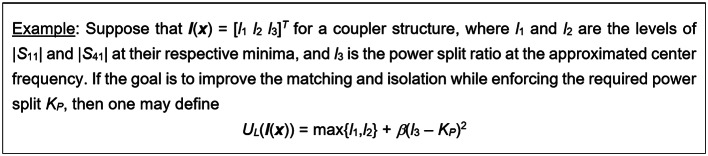
Example of the cost function *U*_*L*_ (cf. (11).

The function *U*_*L*_ is introduced to quantify the circuit performance based on the vector ***l***(***x***). It can be defined as mimicking the original function *U*; see Fig. 5 for an example. However, it does not need to be the equivalent of *U*. At the global search phase, the aim is to push ***f***(***x***) toward ***f***_*t*_. For this purpose, the second factor of (9) is utilized, with *β*_*F*_ being a penalty factor.

#### Global search algorithm

Global search is the first part of the optimization process, which aims to pinpoint the parameter space’s most promising region. Therein, both ***F***(***x***) and ***L***(***x***) are used for prediction of the vectors ***f***(***x***) and ***l***(***x***), and to evaluate the quality of design ***x***. The trial design ***x***_*tmp*_ is generated by minimizing the function *U*_*F*_ of (9) as

10 $${{\bf{x}}_{tmp}} = \arg \mathop {\min }\limits_{{\bf{x}} \in X} {U_F}({\bf{F}}({\bf{x}}),{\bf{L}}({\bf{x}}))$$

The task (10) is confined to the small neighborhood of the simplex {***x***^(*j*)^} by observing the following constraints

11 $$\sum\limits_{j = 1}^n {{a_j} = 1}$$

12 $$- \alpha \leqslant {a_j} \leqslant 1 + \alpha ,\;\;\;j = {\text{ }}1,{\text{ }} \ldots ,n\;\;$$

where *α* > 0 is a small number (here, we use *α* = 0.2), and ***a***(***x***) = [*a*_1_ … *a*_*n*_]^*T*^ are the expansion coefficients (5).

For the sake of implementing the search process, the vectors ***x***^(*j*)^ are organized as follows: ||***f***^(0)^ – ***f***_*t*_|| ≤ ||***f***^(1)^ – ***f***_*t*_|| ≤ … ≤ ||***f***^(*n*)^ – ***f***_*t*_||. As the norm corresponding to ***x***^(0)^ assumes the lowest value, the said vector serves as an initial solution for (10). Having ***x***_*tmp*_, the simplex is updated using the following rules:

*1. Candidate acceptance*: the vector ***x***_*tmp*_ is accepted only if

13 $$\left| {\left| {f\left( {{x_{tmp}}} \right){\text{ }}--{f_t}} \right|} \right|{\text{ }} < {\text{ }}max\{ j \in \left\{ {0,{\text{ }}1,{\text{ }} \ldots ,n} \right\}{\text{ }}:{\text{ }}\left| {\left| {{f^{(j)}}--{f_t}} \right|} \right|\}$$

i.e., it outperforms at least one vertex ***x***^(*j*)^;

*2. Vertex replacement*: if ***x***_*tmp*_ has been accepted, it replaces the vertex ***x***^(*jworst*)^, where

14 $${j_{worst}} = {\text{ }}argmax\{ {j\epsilon}\left\{ {0,{\text{ }}1,{\text{ }} \ldots ,n} \right\}{\text{ }}:{\text{ }}\left| {\left| {{f^{(j)}} - - {f_t}} \right|} \right|\}$$

*3. Simplex reduction*: if ***x***_*tmp*_ has been discarded, the simplex is scaled down toward ***x***^(0)^ as follows:

15 $${{\bf{x}}^{(j)}} \leftarrow \gamma {{\bf{x}}^{(j)}} + (1 - \gamma ){{\bf{x}}^{(0)}}for\;\;\;\;j = {\text{ }}1,{\text{ }} \ldots ,n\;$$

The default value of the reduction factor is *γ* = 0.5. The reduction does not alter the best vertex ***x***^(0)^.

In Sect. 2.4.3, it is shown that reducing the simplex size to a sufficient extent guarantees satisfying condition (13) by ***x***_*tmp*_.

**Fig. 6:**
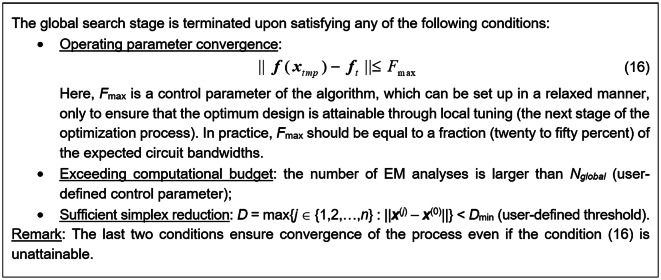
Termination conditions for the global search stage.

The updating procedure is continued until one of the termination criteria, listed in Fig. 6, has been satisfied. Note that termination does not ensure that a design produced by the search procedure satisfies (16); such a design may simply not exist. If this is the case, the procedure returns the best available solution.

#### Enhancement of the objective function

This section shows that reducing the simplex to a sufficient extent ensures that the vector ***x***_*tmp*_ generated by (10) reduces *U*_*F*_ and ||***f***(***x***) – ***f***_*t*_|| w.r.t. their values at ***x***^(0)^, i.e., *U*_*F*_(***f***(***x***_*tmp*_),***l***(***x***_*tmp*_)) < *U*_*F*_(***f***(***x***^(0)^),***l***(***x***^(0)^)) and ||***f***(***x***_*tmp*_) – ***f***_*t*_|| < ||***f***(***x***^(0)^) – ***f***_*t*_||. We start by the following conjecture.

**Conjecture 1:** Assume (at least) a continuous differentiability of the functions ***f***(***x***) and ***l***(***x***) within *X*. Let *D* = max{*j* ∈ {1,2,…,*n*} : ||***x***^(*j*)^ – ***x***^(0)^||} be the simplex size. Then, if *D* is sufficiently small, we have ***F***(***x***) ≈ ***f***(***x***) and ***L***(***x***) ≈ ***l***(***x***), which is understood as ||***F***(***x***) – ***f***(***x***)|| → 0 and ||***L***(***x***) – ***l***(***x***)|| → 0 as *D* → 0.

**Fig. 7:**
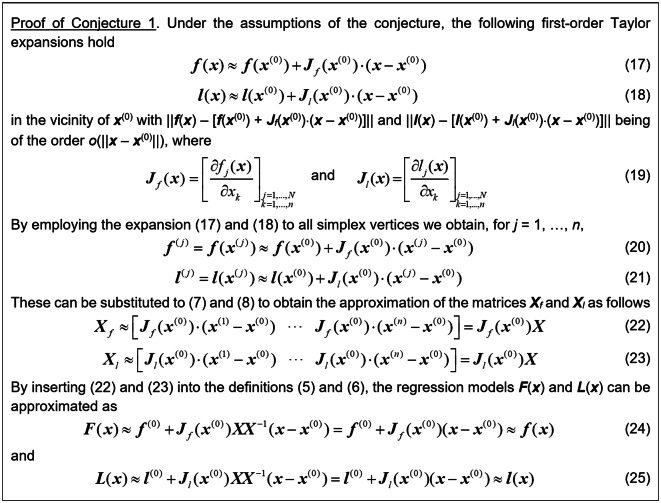
Proof of Conjecture 1.

**Fig. 8:**
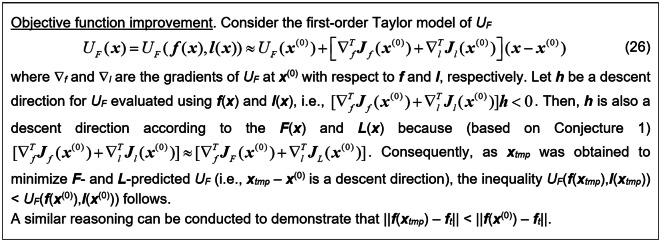
Necessary improvement of the objective function *U*_*F*_ by ***x***_*tmp*_ for small-size simplexes.

The proof of Conjecture 1 can be found in Fig. 7. The alignment of ***F*** and ***f*** (as well as ***L*** and ***l***) implies that at ***x***^(0)^ the matrices ***J***_*F*_ and ***J***_*L*_ (which correspond to the vectors ***F*** and ***L***), are aligned with the sensitivity matrices ***J***_*f*_ and ***J***_*l*_, which correspond to the vectors ***f*** and ***l***. This is sufficient to demonstrate that the vector ***x***_*tmp*_ found by (10) enhances the merit function *U*_*F*_ of (9). The formal proof is provided in Fig. 8.

### Final parameter tuning

The final tuning of the circuit parameters is carried out once the global search stage of Sect. 2.4 has been concluded. This is necessary for two reasons: (i) global optimization was performed using the lower-fidelity EM model, which compromises the reliability, and (ii) global search terminates if the circuit’s operating parameters are aligned with the targets in a sufficient manner, which is not equivalent to minimizing the basic merit function *U*.

Trust-region (TR) gradient-based algorithm^101^ (utilizing the model ***R***_*f*_ of higher accuracy) is employed for the final tuning. In this work, it is accelerated by employing the sparse sensitivity updating strategy of^102^, which is outlined below. The purpose thereof is to limit the number of gradient estimations using finite differentiation^103^, thereby lowering the overall computational cost (see also^104^ for alternative acceleration methods).

We begin by introducing the response variability metric *F*_*v*_. Let. ***R***(***x***) = [*R*(***x***,*f*_1_) … *R*(***x***,*f*_*m*_)]^*T*^, where *f*_*k*_ are the evaluation frequencies. For microwave circuits, the response *R* would normally one of *S*-parameters. We define^102^

16 $${F_v}\left( {{{\bf{R}}_1}({{\bf{x}}_1}),{{\bf{R}}_2}({{\bf{x}}_2})} \right) = \sqrt {\sum\limits_{{f_k} \in F} {{{\left[ {{R_1}({{\bf{x}}_1},{f_k}) - {R_2}({{\bf{x}}_2},{f_k})} \right]}^2}} }$$

where *F* is a considered frequency interval, typically, pertinent to design specifications.

If several responses are considered, e.g., *S*_*k*1_, *k* = 1, …, 4, for a coupler circuit, the metric *F*_*v*_ would be a mean of the metric computed for each characteristic separately^102^.

In the next step, an orthonormal basis of vectors {***v***^(*j*)^}_*j* = 1, …, *n*_, is found. The said vectors ordered with respect to their impact on the variability metric *F*_*v*_. Let ***x***^(*i*)^ be the ongoing iteration point, and ***J***_*R*_(***x***^(*i*)^) be the corresponding sensitivity matrix (Jacobian) of the circuit responses. The first vector ***v***^(1)^ is found by maximizing *F*_*v*_ as

17 $${{\bf{v}}^{(1)}} = \arg \mathop {\max }\limits_{{\bf{v}};\;||{\bf{v}}|| = 1} {F_v}\left( {{{\bf{L}}^{(i)}}({{\bf{x}}^{(i)}} + {\bf{v}}),{\bf{R}}({{\bf{x}}^{(i)}})} \right)$$

In (28), ***L***^(*i*)^ is the first-order Taylor expansion model. The remaining vectors ***v***^(2)^, ***v***^(3)^, …, are found as follows. Given ***v***^(*k*)^, *k* = 1, …, *j*, we solve^102^

18 $${{\bf{v}}^{(j + 1)}} = \arg \mathop {\max }\limits_{{\bf{\bar v}}} {F_v}\left( {{{\bf{L}}^{(i)}}({{\bf{x}}^{(i)}} + {\bf{\bar v}}),{\bf{R}}({{\bf{x}}^{(i)}})} \right)$$

where. The projection *P*^(*j*)^ is defined as ${P^{(j)}}({\bf{v}}) = {\bf{v}} - \sum\nolimits_{k = 1}^j {{{\bf{v}}^{(k)}}\left[ {{{({{\bf{v}}^{(k)}})}^T}{\bf{v}}} \right]}$. Note that the above conditions ensure that ***v***^(*j*+1)^ is of unity lengths and it is orthogonal to all ***v***^(*k*)^, *k* = 1, …, *j*. Also, because ***v***^(*j*+1)^ is searched for in the subspace orthogonal to the previously found directions, we have *F*_*v*_(***L***^(*i*)^(***x***^(*i*)^ + ***v***^(1)^),***R***(***x***^(*i*)^)) > *F*_*v*_(***L***^(*i*)^(***x***^(*i*)^ + ***v***^(2)^),***R***(***x***^(*i*)^)) > … > *F*_*v*_(***L***^(*i*)^(***x***^(*i*)^ + ***v***^(*n*)^),***R***(***x***^(*i*)^)).

Most of the circuit response variability is accounted for the first few directions. To quantify the response variability, we employ *C*_*j*_ factors

19 $${C_j} = \frac{{\sqrt {{{\sum\nolimits_{k = 1}^j {\left[ {{F_v}({{\bf{L}}^{(i)}}({{\bf{x}}^{(i)}} + {{\bf{v}}^{(k)}}),{\bf{R}}({{\bf{x}}^{(i)}}))} \right]} }^2}} }}{{\sqrt {{{\sum\nolimits_{k = 1}^n {\left[ {{F_v}({{\bf{L}}^{(i)}}({{\bf{x}}^{(i)}} + {{\bf{v}}^{(k)}}),{\bf{R}}({{\bf{x}}^{(i)}}))} \right]} }^2}} }}$$

Let *C*_*th*_ refer to a user-defined threshold (here, we use *C*_*th*_ = 0.9, cf^102^. The sensitivity updates are to be restrictive to *N*_*update*_ directions, where

20 $${N_{update}} = \arg \mathop {\min }\limits_{} \left\{ {j \in \{ 1,2,...,n\} :{C_j} \geqslant {C_{th}}} \right\}$$

**Fig. 9:**
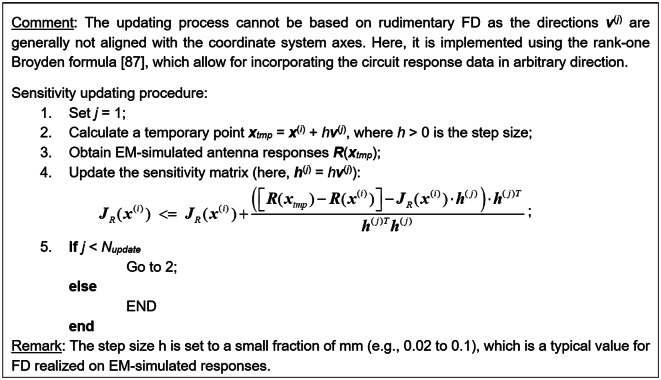
Sensitivity updating by means of principal directions^102,105^.

The sensitivity updating procedure that employs {***v***^(*j*)^}_*j* = 1, …, *Nupdate*_, has been summarized in Fig. 9.

### Optimization framework

**Fig. 10:**
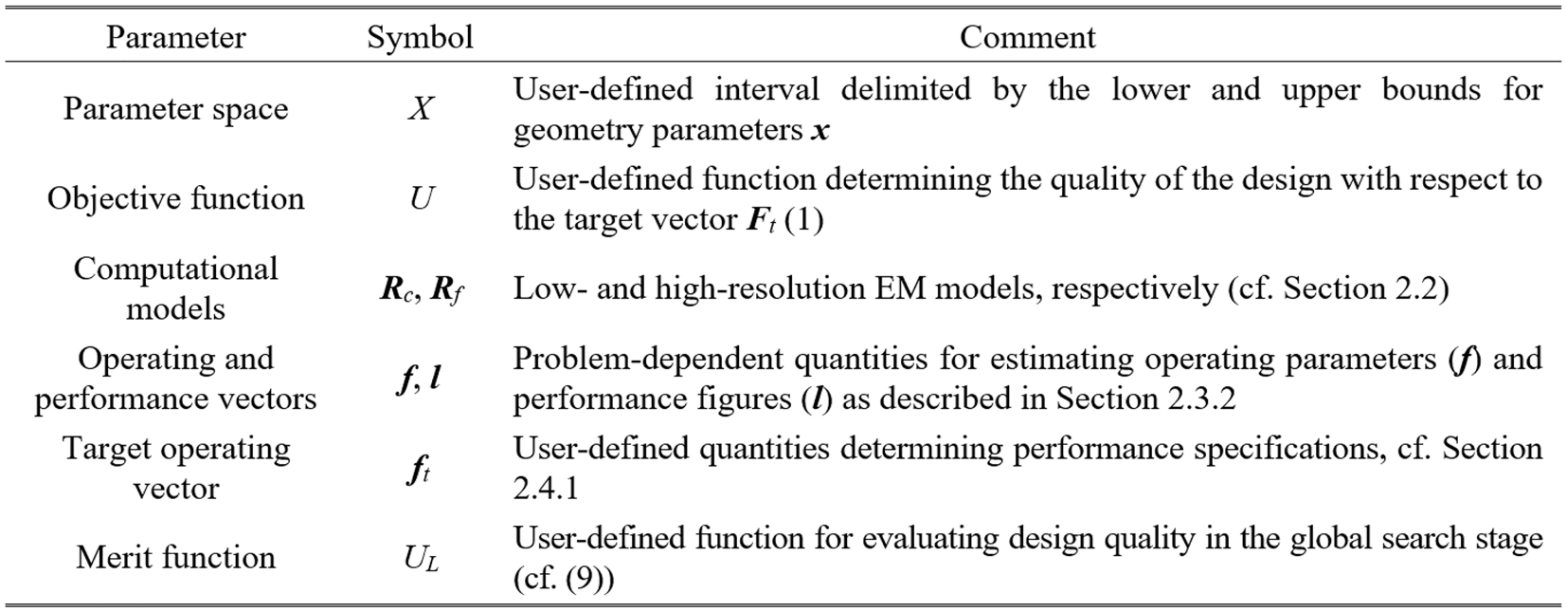
Input parameters of the developed global optimization procedure.

**Fig. 11:**
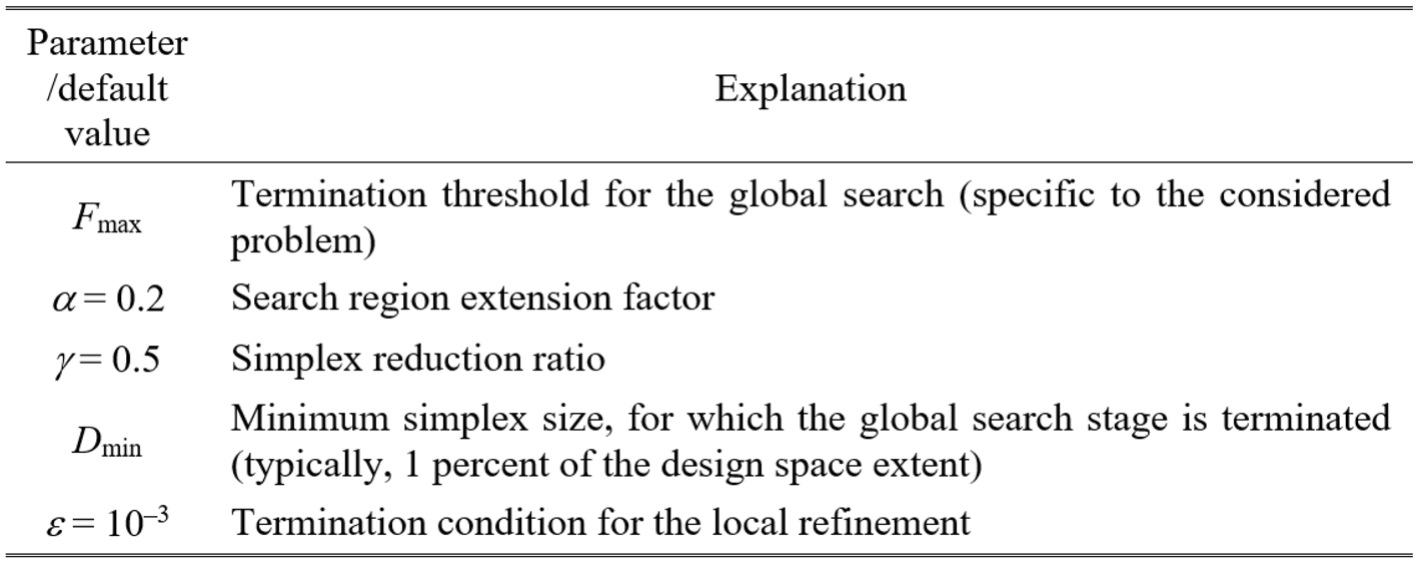
Control parameters of the developed global optimization procedure.

**Fig. 12:**
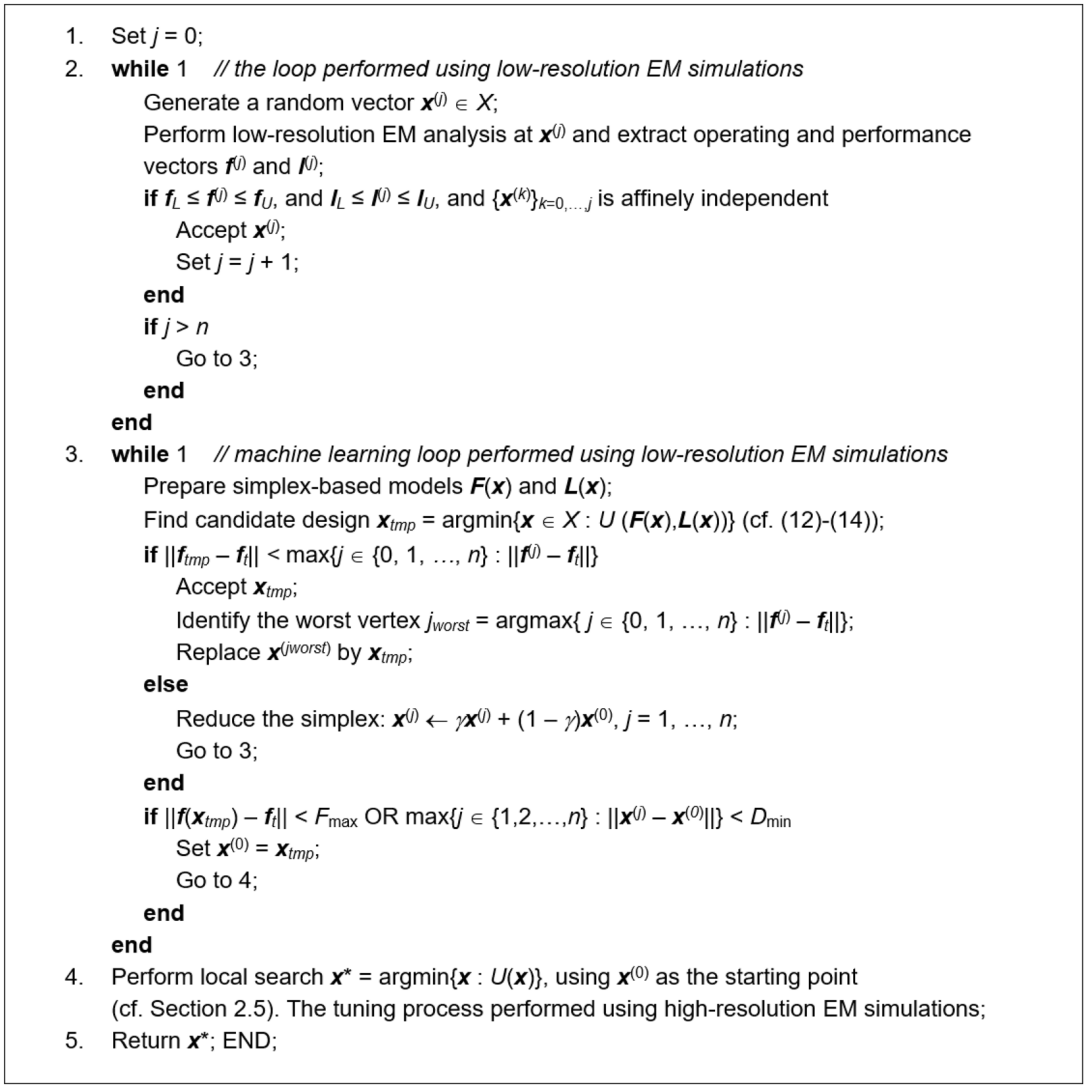
Pseudocode of the global search procedure using simplex-based regressors, dual-resolution EM simulations, and sparse sensitivity updating strategy by means of principal directions.

In this section, we put together the complete optimization framework using the components discussed so far (Sect. 2.1 through 2.5). Figure 10 summarizes the input variables of the algorithm. Figure 11 contains the list of control parameters along with their default values. Figure 12 presents the pseudocode of the optimization procedure.

The performance analysis of the framework presented will be carried out in Sect. 3. Here, we only emphasize that one of the attractive features of the method is easy setup and handling. Among the control parameters of Fig. 11, there is only one which is problem-dependent (cf. Section 2.4.2 for a discussion thereof). The role of the remaining parameters is to govern the search process resolution. Consequently, most of these parameters do not need to be tuned for the optimization task.

At this point, it should be emphasized that the algorithm proposed in this work utilizes mechanisms described earlier in the literature such as simplex-based regression models, response features^99^, variable-fidelity EM simulations^94^, or accelerated gradient-based tuning (here, using principal directions^102^, all these tools are incorporated into a unique algorithmic framework, which exhibits performance superior to methods reported in the literature thus far, as demonstrated in the next section.

It should also be clarified that the proposed algorithm has no connection with physics-based surrogate-assisted approaches such as space mapping (SM) (e.g.,^89,91,92^). Space mapping is normally used for local optimization and exploits an auxiliary low-fidelity (or coarse) model, which, in the case of microwave structures, is typically an equivalent network. The coarse model must undergo an appropriate correction, using auxiliary transformations referred to as input, implicit, or output SM.

The corrected model is then optimized in place of the high-fidelity (or fine) model, and the process is iterated until convergence. The main drawback of SM is that the coarse model is problem-specific and must be developed individually for each system. Also, ensuring its quality and the appropriate choice of SM transformations requires considerable user experience. The algorithm proposed in this work is of a completely different type. It does not rely on any extra models (nor their correction), utilizes simple data-driven models (here, based on simplexes) and is inherently developed to handle global optimization.

It is also important to emphasize the distinction between the proposed approach and conventional machine learning procedures, such as those using kriging, Gaussian process regression (GPR), or Bayesian optimization (which, in fact, is a GPR-based ML). The fundamental difference is the use of simple regressors, along with several other mechanisms combined into an automated design framework, such as pre-screening, dual-resolution EM analysis, and rapid final tuning (which does not only allow for a more precise allocation of the optimum, but also for keeping relaxed termination criteria for the global search stage). The sufficiency of simplex-based regressors is achieved by reformulating the design problem in terms of response features, which flattens the functional landscape to be handled by the algorithm. These features distinguish the proposed approach in a methodological sense from traditional machine learning paradigms and allow for achieving substantially better computational efficiency as illustrated in Sect. 3.

## Validation and performance assessment

Here, the operation of the algorithm outlined in Sect. 2 is showcased using three microstrip circuits. Its performance is compared to five benchmark techniques that include nature-inspired optimization (specifically, the particle swarm optimizer, PSO^106^, and differential evolution, DE^41^, random-start gradient search, and also two machine learning procedures. The latter involve simplex-based predictors but lack acceleration mechanisms present in the proposed technique. The numerical experiments are arranged to verify the optimization process reliability, global search capability, and cost effectiveness of the considered procedures. Section 3.1 delineates the test cases. The setup of the numerical experiments is provided in Sect. 3.2. Section 3.3. provides the results and discusses them.

### Verification test cases

**Fig. 13:**
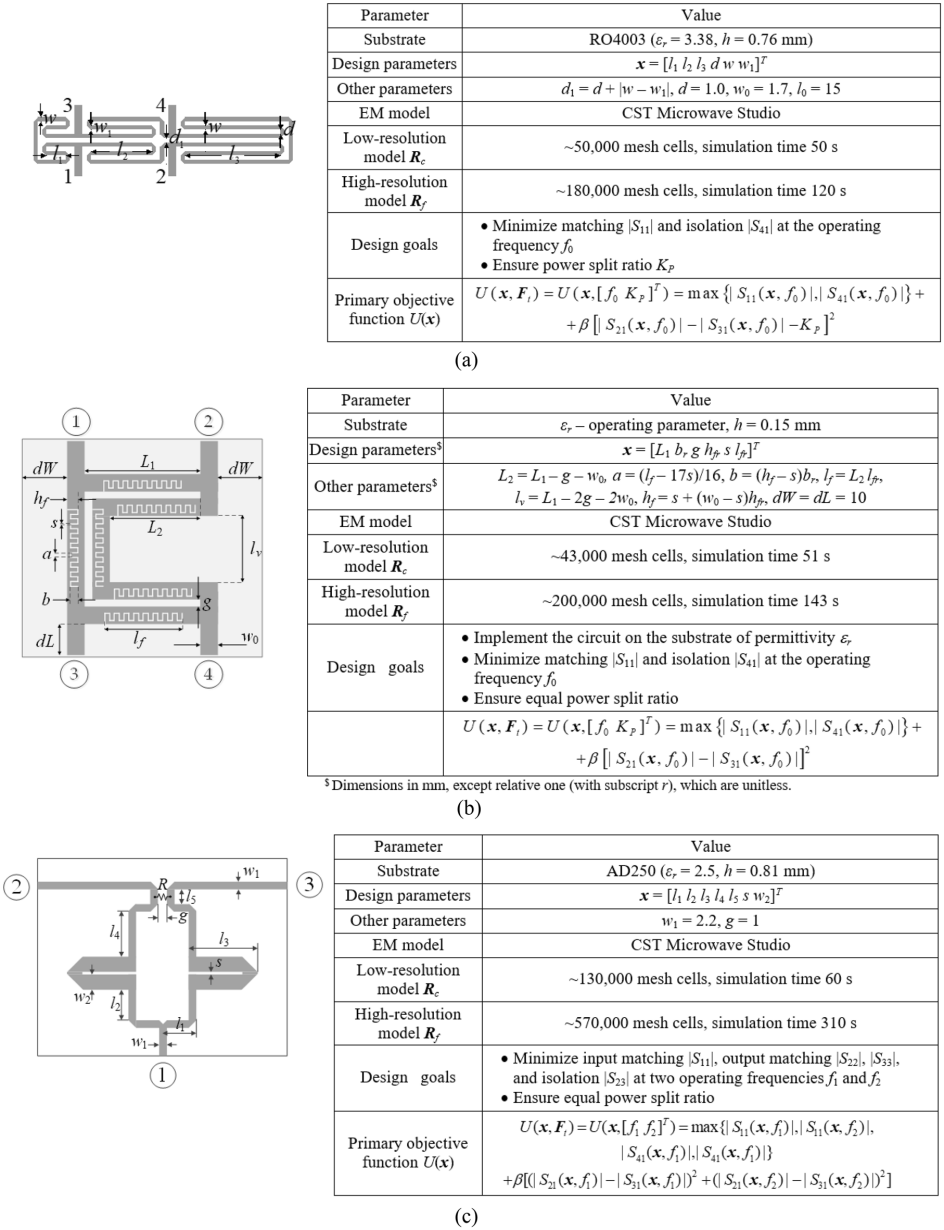
Test circuits: (a) rat-race coupler with folder transmission lines^107^, (b) rat-race coupler coupler with defected microstrip structure^108^, (c) dual-band power divider, *R* denotes a lumped resistor^109^.

The microstrip circuits utilized for verification of our approach are shown in Fig. 13, which also provides essential details concerning their substrates, designable parameters, parameter ranges, and EM models. The design scenarios (two per circuit) have been gathered in Table 1..

### Experimental setup

**Fig. 14:**
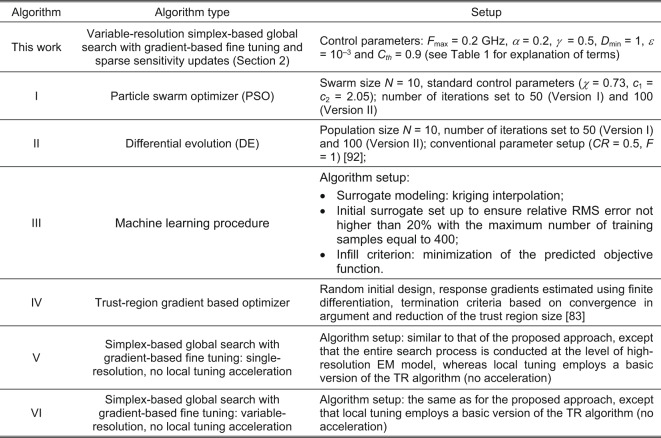
Setup of the introduced procedure and benchmark algorithms.

The test circuits of Fig. 13 were optimized following the scenarios of Table 1. with the use of the proposed framework and six benchmark methods, the details of which can be found in Fig. 14. Identical setup has been employed in all experiments (*F*_max_ = 0.2 GHz, *α* = 0.2, *γ* = 0.5, *D*_min_ = 1, *ε* = 10^–3^, *C*_*th*_ = 0.9), to demonstrate that no tuning of the algorithm to a considered task is necessary. The choice of the benchmark procedures has explicitly been made to emphasize the essential aspects of the approach presented. For example, comparing PSO and DE allows illustrating the computational speedup over population-based metaheuristics, even though a low budget has been used for PSO and DE (only 1,000 objective function calls). Algorithm III is a standard efficient global optimization (EGO)-type machine learning procedure using kriging interpolation models. Comparison with random-start gradient search permits verification of the multimodality of the test problems. Algorithms V and VI, both machine learning routines involving simplex-based surrogates, permit investigating the benefits of incorporating dual-resolution models and restricted sensitivity updates. At the same time, it makes it possible to verify whether these mechanisms deteriorate the design quality.

**Table 1.** Circuits I through III: design goals and spaces.

Circuit	Target operating parameters		Parameter space *X * (lower bounds *l* and upper bounds *u*)
	Symbols	Specific values for numerical experiments	
I	***F***_*t*_ = [*f*_0_ *K*_*P*_]^*T*^	Case 1: *f*_0_ = 1.8 GHz, *K*_*P*_ = − 3 dB	***l*** = [0.5 5.0 5.0 0.2 0.2 0.2]^*T*^***u*** = [15.0 30.0 50.0 2.0 2.0 2.0]^*T*^
		Case 2: *f*_0_ = 1.2 GHz, *K*_*P*_ = 0 dB	
II	***F***_*t*_ = [*f*_0_]^*T*^	Case 1: *f*_0_ = 1.5 GHz, *ε*_*r*_ = 2.5	***l*** = [20.0 0.1 1.0 0.2 0.2 0.2]^*T*^***u*** = [40.0 0.95 5.0 0.95 0.5 0.8]^*T*^
		Case 2: *f*_0_ = 1.2 GHz, *ε*_*r*_ = 4.4	
III	***F***_*t*_ = [*f*_1_ *f*_2_]^*T*^	Case 1: *f*_1_ = 3.0 GHz, *f*_2_ = 4.8 GHz	***l*** = [10.0 1.0 10.0 0.5 1.0 0.1 1.5]^*T*^***u*** = [40.0 20.0 40.0 15.0 6.0 1.5 8.0]^*T*^
		Case 2: *f*_1_ = 2.0 GHz, *f*_2_ = 3.3 GHz	

^$^The circuit is to be optimized for a specific substrate of given relative permittivity e_r_.

## Results and discussion

**Fig. 15:**
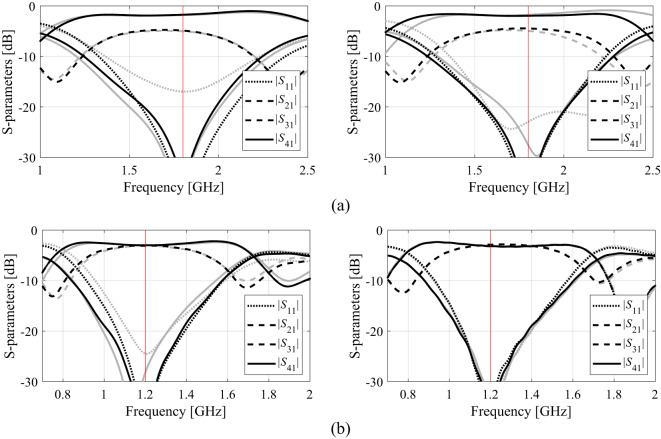
Circuit I: *S*-parameters at optimal designs (black): (a) Case 1, (b) Case 2; along with the responses at the designs found using the global search (gray). The intended operating frequencies are shown in red.

**Fig. 16:**
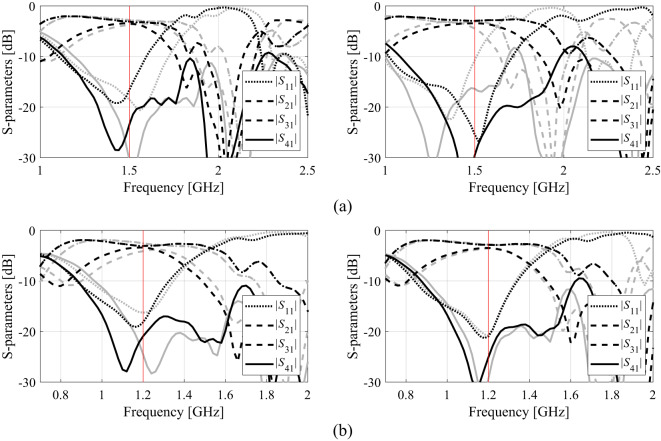
Circuit II: *S*-parameters at optimal designs (black): (a) Case 1, (b) Case 2; along with the responses at the designs found using the global search (gray). The intended operating frequencies are shown in red.

**Fig. 17:**
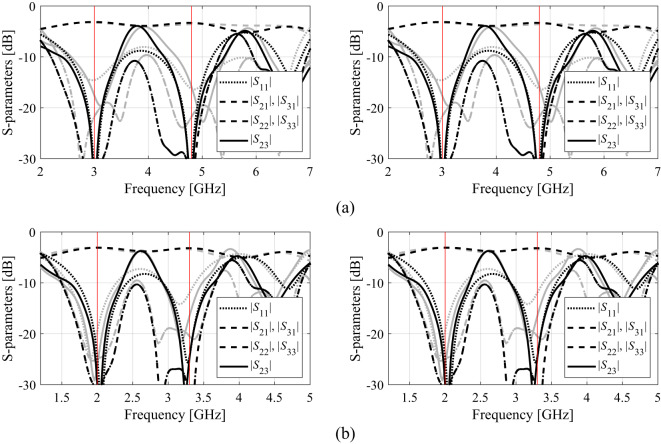
Circuit III: *S*-parameters at optimal designs (black): (a) Case 1, (b) Case 2; along with the responses at the designs found using the global search (gray). The intended operating frequencies are shown in red.

The outcome of our procedure for Circuit I, II, and III has been presented in Tables 2 and 3, and Table 4. Table 5 provides the average CPU times (in hours) for the proposed algorithm and all benchmark methods. Figures 15 and 16, and 17 show the circuit responses obtained for certain runs of the proposed algorithm. For each test case and each design scenario, the procedure of Sect. 2 and all benchmark algorithms underwent ten runs.

The tables contain the average values of the objective function, the running cost (recalculated w.r.t. the number of high-fidelity EM analyses), and the success rate (i.e., the number of runs where the circuit operating parameters were placed to ensure ||***f***(x^*^) – ***f***_*t*_) ≤ *F*_max_). Regarding the cost, Tables 2, 3 and 4 also provide the minimum and maximum expenses for all methods considered. In the following paragraphs, they analyze the performance of the proposed framework in comparison to the benchmark methods in terms of reliability, quality of the produced designs, and computational efficiency.

The first performance indicator that we investigate is reliability. As announced earlier, the metric utilized for this purpose is the success rate, defined as a relative number of optimization runs leading to a design meeting the condition ||***f***(x^*^) – ***f***_*t*_)|| ≤ *F*_max_. A quick comparison with random-start gradient search corroborates the multimodality of all test problems: the success rate for this method is only 5/10 on average.

**Table 2:** Circuit I: optimization results and benchmarking.

Optimization method		Case 1			Case 2		
		Average merit function [dB]	Optimization cost^$^	Success rate^#^	Average merit function [dB]	Optimization cost^$^	Success rate^#^
Dual-resolution simplex-based algorithm with sparse sensitivity updates (this work)		–33.9	48.9–54.3–61.3	10/10	–37.7	33.7–38.2–42.4	10/10
Algorithm I (PSO)	Version I (50 iterations)	–24.8	500–500–500	9/10	–23.7	500–500–500	9/10
	Version II (100 Iterations)	–34.0	1000–1,000–1000	10/10	–36.2	1000–1,000–1000	10/10
Algorithm II (DE)	Version I (50 iterations)	–22.8	500–500–500	8/10	–25.8	500–500–500	9/10
	Version II (100 Iterations)	–33.2	1000–1000–1000	10/10	–35.6	1000–1,000–1000	10/10
Algorithm III (machine learning)		–31.5	435–468.3–502	10/10	–34.8	473–485.3–510	10/10
Algorithm IV (Trust-region algorithm)		–18.7	72–102.8–123	6/10	48.3	62–68.7–73	5/10
Algorithm V (Simplex-based algorithm with high-fidelity model; no local tuning acceleration)		–33.6	62–74.6–83	10/10	–38.3	48–55.7–61	10/10
Algorithm VI (Simplex-based algorithm with dual-resolution EM models, no local tuning acceleration)		–32.9	54.2–60.2–65.8	10/10	–37.5	40.2–43.1–46.3	10/10

^$^The cost is represented by the number of higher-resolution EM simulations of the considered device. Shown are minimum, average, and maximum values.

^#^Number of procedure executions for which satisfactory allocation of the operating frequencies has been accomplished.

**Table 3:** Circuit II: optimization results and benchmarking.

Optimization method		Case 1			Case 2		
		Average merit function [dB]	Optimization cost^$^	Success rate^#^	Average merit function [dB]	Optimization cost^$^	Success rate^#^
Dual-resolution simplex-based algorithm with sparse sensitivity updates (this work)		–18.5	46.5–49.7–52.3	10/10	–20.5	38.4–41.4–45.6	10/10
Algorithm I (PSO)	Version I (50 iterations)	–17.6	500–500–500	10/10	–19.4	500–500–500	9/10
	Version II (100 Iterations)	–19.2	1000–1,000–1000	10/10	–22.5	1000–1,000–1000	10/10
Algorithm II (DE)	Version I (50 iterations)	–17.9	500–500–500	9/10	–18.9	500–500–500	9/10
	Version II (100 Iterations)	–18.3	1000–1,000–1000	10/10	–21.5	1000–1,000–1000	10/10
Algorithm III (machine learning)		–18.1	420–453.2–490	10/10	–19.5	451–473.4–499	10/10
Algorithm III (Trust-region algorithm)		1.8	72–77.0–83	5/10	7.6	78–83.8–91	5/10
Algorithm IV (Simplex-based algorithm with high-fidelity model; no local tuning acceleration)		–18.6	71–76.9–84	10/10	–19.4	61–69.8–75	10/10
Algorithm V (Simplex-based algorithm with dual-resolution EM models, no local tuning acceleration)		–18.2	58.4–63.1–69.2	10/10	–18.5	49.5–55.8–61.2	10/10

$ The cost is represented by the number of higher-resolution EM simulations of the considered device. Shown are minimum, average, and maximum values.# Number of procedure executions for which satisfactory allocation of the operating frequencies has been accomplished.

**Table 4:** Circuit III: optimization results and benchmarking.

Optimization method		Case 1			Case 2		
		Average merit function [dB]	Optimization cost^$^	Success rate^#^	Average merit function [dB]	Optimization cost^$^	Success rate^#^
Dual-resolution simplex-based algorithm with sparse sensitivity updates (this work)		–33.9	43.5–47.4–49.9	10/10	–25.1	33.5–37.0–41.2	10/10
Algorithm I (PSO)	Version I (50 iterations)	–19.6	500–500–500	8/10	–18.8	500–500–500	8/10
	Version II (100 Iterations)	–18.8	1000–1,000–1000	9/10	–19.7	1000–1,000–1000	9/10
Algorithm II (DE)	Version I (50 iterations)	–19.9	500–500–500	9/10	–20.0	500–500–500	8/10
	Version II (100 Iterations)	–21.5	1000–1,000–1000	9/10	–20.0	1000–1,000–1000	9/10
Algorithm III (machine learning)		–29.5	425–463.4–499	9/10	–24.3	434–461.5–505	10/10
Algorithm IV (Trust-region algorithm)		–12.3	89–95.1–101	2/10	–20.6	85–93.8–100	7/10
Algorithm V (Simplex-based algorithm with high-fidelity model; no local tuning acceleration)		–33.8	75–82.0–92	10/10	–25.9	78–84.6–93	10/10
Algorithm VI (Simplex-based algorithm with dual-resolution EM models, no local tuning acceleration)		–34.9	55.4–60.6–65.8	10/10	–25.8	58.3–62.7–69.4	10/10

$ The cost is represented by the number of higher-resolution EM simulations of the considered device. Shown are minimum, average, and maximum values.# Number of procedure executions for which satisfactory allocation of the operating frequencies has been accomplished.

**Table 5:** Optimization costs for circuits I through III: average CPU times in hours and standard deviation of the objective function value in dB.

Optimization method		Circuit I		Circuit II		Circuit III	
		Case 1	Case 2	Case 1	Case 2	Case 3	Case 4
Dual-resolution simplex-based algorithm with sparse sensitivity updates (this work)		1.8 h [4.4 dB]	1.3 h [5.5 dB]	2.0 h [2.7 dB]	1.6 h [2.9 dB]	4.1 h [3.0 dB]	3.2 h [3.5 dB]
Algorithm I (PSO)	Version I (50 iterations)	16.7 h 8.2 dB]	16.7 h [8.5 dB]	19.9 h [5.2 dB]	19.9 h [6.2 dB]	43.1 h [8.0 dB}	43.1 h [6.9 dB]
	Version II (100 Iterations)	33.3 h 6.8 dB]	33.3 h [7.8 dB]	39.7 h [4.3 dB]	39.7 h [5.5 dB]	86.1 h [7.2 dB]	86.1 h [6.1 dB]
Algorithm II (DE)	Version I (50 iterations)	16.7 h [7.9 dB]	16.7 h [7.5 dB]	19.9 h [4.9 dB]	19.9 h [6.0 dB]	43.1 h [7.8 dB]	43.1 h [7.0 dB]
	Version II (100 Iterations)	33.3 h [7.5 dB]	33.3 h [7.0 dB]	39.7 h [4.5 dB]	39.7 h [5.3 dB]	86.1 h [6.9 dB]	86.1 h [6.2 dB]
Algorithm III		15.6 h [10.2 dB]	16.2 h [12.8 dB]	18.0 h [3.3 dB]	18.8 h [4.0 dB]	41.9 h [6.1 dB]	39.7 h [5.8 dB]
Algorithm IV		3.4 h [5.2 dB]	2.3 h [6.0 dB]	3.1 h [3.0 dB]	3.3 h [3.5 dB]	8.2 h [3.5 dB]	8.1 h [3.9 dB]
Algorithm V		2.5 h [5.0 dB]	1.9 h [5.6 dB]	3.1 h [3.1 dB]	2.8 h [3.3 dB]	7.1 h [3.2 dB]	7.3 h [3.5 dB]
Algorithm VI		2.0 h [5.0 dB]	1.4 h [5.3 dB]	2.5 h [2.8 dB]	2.2 h [3.2 dB]	5.2 h [3.6 dB]	5.4 h [3.4 dB]

Population-based optimization turns out to be considerably better; however, for the budget of 500 function calls, it is still not perfect (8.7/10). For the budget of 1,000 it is closer to perfect, namely 9.7/10. On the other hand, both the proposed procedure and the machine learning techniques (Algorithms III, V and VI), exhibit 10/10 success rate. Recall, that all these methods except for Algorithm III share the same underlying principles in terms of the global search mechanism. Clearly, the technique introduced in this work is considerably faster, which will be elaborated below. The conventional EGO-based method (Algorithm III) yields the results of similar quality as the proposed approach; however, it is considerably slower due to the necessity of building a data-driven surrogate across parameter space.

As for the design quality, which is quantified using the average value of the merit function, the proposed method is comparable to all global optimization procedures in the benchmark set (Algorithms I, II, III, V, and VI), except Circuit III, where nature-inspired optimization produced noticeable inferior results. Also, significant differences between PSO results obtained for Version I and II (500 and 1,000 function calls, respectively) is indicative of insufficient computational budget. The latter was intentionally set to these numbers to avoid excessive running times, which are still up to three CPU days for some of the problems. Observe that differences of magnitude a few decibels are insignificant for function values below − 25 dB or so.

Perhaps the major advantage of the proposed optimization technique is its exceptional cost-efficiency. The overall cost (averaged across Circuits I through II and all considered design tasks) is only about 45 EM simulations (of higher resolution). This is almost 50% less than the cost of a direct gradient-based search at a high-fidelity level, which is unsurprising. Although the proposed technique contains gradient-based search as its last stage, it is executed from good starting points produced by the machine learning process and expedited by employing the sparse sensitivity updating scheme. The savings over Algorithm I (PSO) and Algorithm II (DE) equal 96%, meaning that our procedure is over twenty times faster than nature-inspired optimization. In addition, our approach is almost 90% faster than the EGO-based method. Moreover, the effects of including dual-fidelity simulations and sparse sensitivity updates lead to about 40% speedup (i.e., versus Algorithm IV), whereas the latter alone brings about 20% acceleration (i.e., versus Algorithm V). Meanwhile, the mentioned computational advantages are achieved without degrading the design quality.

Altogether, it can be concluded that all the algorithmic components integrated into the presented framework are relevant to ensure the desired properties of the optimization process: reliability and low running cost. These include conducting the search process from standpoint of the operating parameters of the circuits and using structurally simple surrogates, usage of dual-fidelity models, and lowering the number of FD-based sensitivity updates in the final tuning phase. Further, the number of control parameters is small, and they do not need to be tuned to a range of practical design tasks, which makes the procedure easy to handle.

## Conclusion

This paper put forward a resource-efficient technique for global optimization of microwave passives. The search process targets the operating parameters of the considered circuit, which facilitates the identification of the conducive portions of the parameter space, and enables the utilization of structurally simple surrogate models. The machine learning procedure implemented using these tools is provably convergent and executed using a low-fidelity simulation model to achieve computational speedup. Reliability is ensured by a complementary gradient-based parameter tuning carried out using high-fidelity models but also involving a sparse sensitivity updating strategy to reduce the running costs even further.

Extensive verification experiments involving three microstrip structures and two design scenarios per circuit conclusively demonstrated the competitive performance of our algorithm. On the one hand, it ensured a perfect success rate with high-quality designs found in all optimization runs. On the other hand, the overall cost of our procedure is exceptionally low and equals less than fifty EM simulations at high-fidelity levels on average. This is considerably less than direct gradient-based search and corresponds to almost 96% savings over nature-inspired optimization. At the same time, the incorporation of dual-fidelity models and restricted sensitivity updates result in about 20% speedup each. The savings mentioned are not followed by any deterioration in the design quality.

Despite the merits discussed, the presented approach has a potential limitation, which may emerge in the case of excessively large search spaces. If the ranges of geometry parameters are very broad, most of the randomly sampled designs would be of poor quality, which impairs the pre-sampling procedure described in Sect. 2.3.2, the goal of which was to identify a set of affinely independent points to be used as the initial simplex vertices. Inferior designs would be repetitively rejected as their corresponding operating parameters are impossible to extract (e.g., may not exist, or be outside the range of interest), which inflates the overall computational cost. Still, the likelihood of this situation occurring is not so high if the definition of the parameter space has been cautious and/or initial parametric studies of the circuit at hand. Of course, large parameter spaces would be problematic for any global optimization algorithm just as well. A part of the future work will be further investigation of this issue. With this reservation, the findings of the paper indicate that our framework might be suitable as an alternative to the existing global search procedures, both nature-inspired routines and machine learning methods processing the complete frequency responses, although more extensive numerical studies involving a broader range of test cases are necessary to support this claim.

Future work will be focused on extending the applicability of the proposed algorithm to other types of high-frequency systems such as filters, metamaterial unit cells, and antennas. Another topic of further research will be investigating the properties of the algorithm for more complex problems, especially, higher-dimensionality parameter spaces.

## Data Availability

The datasets used and/or analyzed during the current study are available from the corresponding author on reasonable request.

## References

[1] Ma, P. et al. „A design method of multimode multiband bandpass filters. *IEEE Trans. Microw. Theory Techn*. **66** (6), 2791–2799 (2018).

[2] Yang, Q., Jiao, Y. & Zhang, Z. „Compact multiband bandpass filter using low-pass filter combined with open stub-loaded shorted stub. *IEEE Trans. Microw. Theory Techn*. **66** (4), 1926–1938 (2018).

[3] Hagag, M. F., Zhang, R. & Peroulis, D. „High-performance tunable narrowband SIW cavity-based quadrature hybrid coupler. *IEEE Microw. Wirel. Comp. Lett.* **29** (1), 41–43 (2019).

[4] Gómez-García, R., Rosario-De, J., Jesus & Psychogiou, D. „Multi-band bandpass and Bandstop RF filtering couplers with dynamically-controlled bands. *IEEE Access.* **6**, 32321–32327 (2018).

[5] Zhang, R. & Peroulis, D. „Mixed lumped and distributed circuits in wideband bandpass filter application for spurious-response suppression. *IEEE Microw. Wirel. Comp. Lett.* **28** (11), 978–980 (2018).

[6] Liu, H., Fang, S., Wang, Z. & Fu, S. „Design of arbitrary-phase-difference transdirectional coupler and its application to a flexible butler matrix. *IEEE Trans. Microw. Theory Techn*. **67** (10), 4175–4185 (2019).

[7] Li, Q., Chen, X., Chi, P. & Yang, T. „Tunable Bandstop filter using distributed coupling microstrip resonators with capacitive terminal. *IEEE Microw. Wirel. Comp. Lett.* **30** (1), 35–38 (2020).

[8] Sheikhi, A., Alipour, A. & Mir, A. „Design and fabrication of an ultra-wide stopband compact bandpass filter. *IEEE Trans. Circuits Syst. II: Express Briefs*. **67** (2), 265–269 (2020).

[9] Firmansyah, T., Alaydrus, M., Wahyu, Y., Rahardjo, E. T. & Wibisono, G. A highly independent multiband bandpass filter using a multi-coupled line stub-SIR with folding structure. *IEEE Access.* **8**, 83009–83026 (2020).

[10] Chen, S. et al. A frequency synthesizer based microwave permittivity sensor using CMRC structure. *IEEE Access.* **6**, 8556–8563 (2018).

[11] Chi, J. G. & Kim, Y. J. „A compact wideband millimeter-wave quadrature hybrid coupler using artificial transmission lines on a glass substrate. *IEEE Microw. Wirel. Comp. Lett.* **30** (11), 1037–1040 (2020).

[12] Deng, J., Li, M., Sun, D., Guo, L. & Ma, X. „Compact dual-band inverted-microstrip ridge gap waveguide bandpass filter. *IEEE Trans. Microw. Theory Techn*. **68** (7), 2625–2632 (2020).

[13] Kurgan, P. & Koziel, S. Selection of circuit geometry for miniaturized microwave components based on concurrent optimization of performance and layout area. *AEU – Int. J. Electr. Comm.* **108**, 287–294 (2019).

[14] Koziel, S. & Abdullah, M. Machine-learning-powered EM-based framework for efficient and reliable design of low scattering metasurfaces. *IEEE Trans. Microw. Theory Techn*. **69** (4), 2028–2041 (2021).

[15] Li, Y., Ren, P. & Xiang, Z. „A dual-passband frequency selective surface for 5G communication. *IEEE Antennas Wirel. Propag. Lett.* **18** (12), 2597–2601 (2019).

[16] Li, H., Jiang, Y., Ding, Y., Tan, J. & Zhou, J. Low-sidelobe pattern synthesis for sparse conformal arrays based on PSO-SOCP optimization. *IEEE Access.* **6**, 77429–77439 (2018).

[17] Rayas-Sanchez, J. E., Koziel, S. & Bandler, J. W. Advanced RF and microwave design optimization: a journey and a vision of future trends. *IEEE J. Microwaves*. **1** (1), 481–493 (2021).

[18] Jin, H., Zhou, Y., Huang, Y. M., Ding, S. & Wu, K. Miniaturized broadband coupler made of slow-wave half-mode substrate integrated waveguide. *IEEE Microw. Wirel. Comp. Lett.* **27** (2), 132–134 (2017).

[19] Pietrenko-Dabrowska, A. & Koziel, S. Fast design closure of compact microwave components by means of feature-based metamodels. *Electronics* **10**(1), 10 (2021).

[20] Li, X. & Luk, K. M. The grey Wolf optimizer and its applications in electromagnetics. *IEEE Trans. Ant Prop.* **68** (3), 2186–2197 (2020).

[21] Luo, X., Yang, B. & Qian, H. J. Adaptive synthesis for resonator-coupled filters based on particle swarm optimization. *IEEE Trans. Microw. Theory Techn*. **67** (2), 712–725 (2019).

[22] Majumder, A., Chatterjee, S., Chatterjee, S., Sinha Chaudhari, S. & Poddar, D. R. Optimization of small-signal model of GaN HEMT by using evolutionary algorithms. *IEEE Microw. Wirel. Comp. Lett.* **27** (4), 362–364 (2017).

[23] Choi, K. et al. Hybrid algorithm combing genetic algorithm with evolution strategy for antenna design. *IEEE Trans. Magn* **52**(3), 1–4 (2016) (**Art 7209004**).

[24] Ghorbaninejad, H. & Heydarian, R. New design of waveguide directional coupler using genetic algorithm. *IEEE Microw. Wirel. Comp. Lett.* **26** (2), 86–88 (2016).

[25] Zhu, D. Z., Werner, P. L. & Werner, D. H. Design and optimization of 3-D frequency-selective surfaces based on a multiobjective lazy ant colony optimization algorithm. *IEEE Trans. Ant Propag.* **65** (12), 7137–7149 (2017).

[26] Ding, D., Zhang, Q., Xia, J., Zhou, A. & Yang, L. Wiggly parallel-coupled line design by using multiobjective evolutionay algorithm. *IEEE Microw. Wirel. Comp. Lett.* **28** (8), 648–650 (2018).

[27] Greda, L. A., Winterstein, A., Lemes, D. L. & Heckler, M. V. T. Beamsteering and beamshaping using a linear antenna array based on particle swarm optimization. *IEEE Access.* **7**, 141562–141573 (2019).

[28] Cui, C., Jiao, Y. & Zhang, L. „Synthesis of some low sidelobe linear arrays using hybrid differential evolution algorithm integrated with convex programming. *IEEE Ant Wirel. Propag. Lett.* **16**, 2444–2448 (2017).

[29] Baumgartner, P. et al. Multi-objective optimization of Yagi-Uda antenna applying enhanced firefly algorithm with adaptive cost function. *IEEE Trans. Magnetics*. **54** (3), 8000504 (2018).

[30] Yang, S. H. & Kiang, J. F. Optimization of sparse linear arrays using harmony search algorithms. *IEEE Trans. Ant Prop.* **63** (11), 4732–4738 (2015).

[31] Li, X. & Guo, Y. X. Multiobjective optimization design of aperture illuminations for microwave power transmission via multiobjective grey Wolf optimizer. *IEEE Trans. Ant Prop.* **68** (8), 6265–6276 (2020).

[32] Zheng, T. et al. IWORMLF: improved invasive weed optimization with random mutation and Lévy flight for beam pattern optimizations of linear and circular antenna arrays. *IEEE Access.* **8**, 19460–19478 (2020).

[33] Al-Azza, A. A., Al-Jodah, A. A. & Harackiewicz, F. J. Spider monkey optimization: a novel technique for antenna optimization. *IEEE Antennas Wirel. Propag. Lett.* **15**, 1016–1019 (2016).

[34] Liang, S. et al. „Sidelobe reductions of antenna arrays via an improved chicken swarm optimization approach. *IEEE Access.* **8**, 37664–37683 (2020).

[35] Li, W., Zhang, Y. & Shi, X. „Advanced fruit fly optimization algorithm and its application to irregular subarray phased array antenna synthesis. *IEEE Access.* **7**, 165583–165596 (2019).

[36] Jiang, Z. J., Zhao, S., Chen, Y. & Cui, T. J. „Beamforming optimization for time-modulated circular-aperture grid array with DE algorithm. *IEEE Ant Wirel. Propag. Lett.* **17** (12), 2434–2438 (2018).

[37] Bayraktar, Z., Komurcu, M., Bossard, J. A. & Werner, D. H. The wind driven optimization technique and its application in electromagnetics. *IEEE Trans. Antennas Propag.* **61** (5), 2745–2757 (2013).

[38] Rayno, J., Iskander, M. F. & Kobayashi, M. H. Hybrid genetic programming with accelerating genetic algorithm optimizer for 3-D metamaterial design. *IEEE Antennas Wirel. Propag. Lett.* **15**, 1743–1746 (2016).

[39] Abdelhafiz, A., Behjat, L. & Ghannouchi, F. M. Generalized memory polynomial model dimension selection using particle swarm optimization. *IEEE Microw. Wirel. Comp. Lett.* **28** (2), 96–98 (2018).

[40] Goudos, S. K., Yioultsis, T. V., Boursianis, A. D., Psannis, K. E. & Siakavara, K. Application of new hybrid Jaya grey Wolf optimizer to antenna design for 5G communications systems. *IEEE Access.* **7**, 71061–71071 (2019).

[41] Liu, F., Liu, Y., Han, F., Ban, Y. & Jay Guo, Y. Synthesis of large unequally spaced planar arrays utilizing differential evolution with new encoding mechanism and cauchy mutation. *IEEE Trans. Antennas Propag.* **68** (6), 4406–4416 (2020).

[42] Karimkashi, S. & Kishk, A. A. Invasive weed optimization and its features in electromagnetics. *IEEE Trans. Antennas Propag.* **58** (4), 1269–1278 (2010).

[43] Kovaleva, M., Bulger, D. & Esselle, K. P. „Comparative study of optimization algorithms on the design of broadband antennas. *IEEE J. Multiscale Multiphysics Comp. Techn*. **5**, 89–98 (2020).

[44] Bai, Y., Xiao, S., Liu, C. & Wang, B. A hybrid IWO/PSO algorithm for pattern synthesis of conformal phased arrays. *IEEE Trans. Antennas Propag.* **61** (4), 2328–2332 (2013).

[45] Li, Y., Xiao, S., Rotaru, M. & Sykulski, J. K. A dual kriging approach with improved points selection algorithm for memory efficient surrogate optimization in electromagnetics. *IEEE Trans. Magn* **52**(3), 1–4 (2016) (**Art 7000504**).

[46] Ogut, M., Bosch-Lluis, X. & Reising, S. C. „A deep learning approach for microwave and millimeter-wave radiometer calibration. *IEEE Trans. Geoscience Remote Sens.* **57** (8), 5344–5355 (2019).

[47] Jacobs, J. P. Characterization by Gaussian processes of finite substrate size effects on gain patterns of microstrip antennas. *IET Microwaves Ant Prop.* **10** (11), 1189–1195 (2016).

[48] Zhang, Z., Cheng, Q. S., Chen, H. & Jiang, F. An efficient hybrid sampling method for neural network-based microwave component modeling and optimization. *IEEE Microw. Wirel. Comp. Lett.* **30** (7), 625–628 (2020).

[49] Van Nechel, E., Ferranti, F., Rolain, Y. & Lataire, J. Model-driven design of microwave filters based on scalable circuit models. *IEEE Trans. Microw. Theory Techn*. **66** (10), 4390–4396 (2018).

[50] Yu, X. et al. „A method to select optimal deep neural network model for power amplifiers. *IEEE Microw. Wirel. Comp. Lett.* **31** (2), 145–148 (2021).

[51] Na, W. et al. „Advanced extrapolation technique for neural-based microwave modeling and design. *IEEE Trans. Microw. Theory Techn*. **66** (10), 4397–4418 (2018).

[52] Petrocchi, A. et al. „Measurement uncertainty propagation in transistor model parameters via polynomial chaos expansion. *IEEE Microw. Wirel. Comp. Lett.* **27** (6), 572–574 (2017).

[53] Couckuyt, I., Declercq, F., Dhaene, T., Rogier, H. & Knockaert, L. Surrogate-based infill optimization applied to electromagnetic problems. *Int. J. RF Microw. Computt -Aided Eng.* **20** (5), 492–501 (2010).

[54] Torun, H. M. & Swaminathan, M. High-dimensional global optimization method for high-frequency electronic design. *IEEE Trans. Microw. Theory Techn*. **67** (6), 2128–2142 (2019).

[55] Liu, B., Koziel, S. & Zhang, Q. A multi-fidelity surrogate-model-assisted evolutionary algorithm for computationally expensive optimization problems. *J. Comp. Sc*. **12**, 28–37 (2016).

[56] Sun, Y. et al. Data-driven bayesian optimization framework for rapidly developing novel wideband, low-profile dipole antenna with 3-D-printed technology. *IEEE Trans. Ant Propag.* **73** (1), 108–120 (2025).

[57] Mwang’amba, R., Mei, P., Akinsolu, M. O., Liu, B. & Zhang, S. Gain bandwidth enhancement and sidelobe level stabilization of Mmwave lens antennas using AI-driven optimization. *IEEE Ant Wirel. Propag. Lett.* **23** (11), 3554–3558 (2024).

[58] Liu, Y. et al. An efficient method for antenna design based on a self-adaptive bayesian neural network-assisted global optimization technique. *IEEE Trans. Ant Propag.* **70** (12), 11375–11388 (2022).

[59] Gao, T. Y., Jiao, Y. C., Zhang, Y. X. & Zhang, L. A hybrid self-adaptive differential evolution algorithm with simplified bayesian local optimizer for efficient design of antennas. *IEEE Trans. Ant Propag.* **73** (1), 391–404 (2025).

[60] Li, J., Yang, A., Tian, C., Ye, L. & Chen, B. Multi-fidelity bayesian algorithm for antenna optimization. *J. Syst. Eng. Electr.* **33** (6), 1119–1126 (2022).

[61] Wei, Z. et al. Automated antenna design via domain knowledge-informed reinforcement learning and imitation learning. *IEEE Trans. Ant Propag.* **71** (7), 5549–5557 (2023).

[62] Wu, Z. M. et al. Design of wideband microstrip-to-microstrip vertical transition with pixel structures based on reinforcement learning. *IEEE Microw. Wirel. Comp. Lett.* **35** (3), 274–277 (2025).

[63] Peng, F. & Chen, X. An antenna optimization framework based on deep reinforcement learning. *IEEE Trans. Ant Propag.* **72** (10), 7594–7605 (2024).

[64] Pan, J. H. et al. Deep reinforcement learning based optimization of microwave microfluidic sensor. *IEEE Microw. Wirel. Techn Lett.* **34** (11), 1309–1312 (2024).

[65] Na, W. et al. Efficient EM optimization exploiting parallel local sampling strategy and bayesian optimization for microwave applications. *IEEE Microw. Wirel. Comp. Lett.* **31** (10), 1103–1106 (2021).

[66] Swaminathan, M., Bhatti, O. W., Guo, Y., Huang, E. & Akinwande, O. Bayesian learning for uncertainty quantification, optimization, and inverse design. *IEEE Microwave Wireless Comp. Lett 31*(10), 274–277 (2022).

[67] Cui, J. et al. Advanced Bayesian-inspired multilayer effective parameter determination method for automated ANN model generation of microwave components. *IEEE Trans. Microw. Theory Techn*. **72** (8), 4408–4420 (2024).

[68] Zhou, Z. et al. Bayesian-inspired sampling for efficient machine-learning-assisted microwave component design. *IEEE Trans. Microw. Theory Techn*. **72** (2), 996–1007 (2024).

[69] Li, S., Alsalman, O., Huang, J. & Zhu, C. In-plane and out-of-plane 2-D microdisplacement sensor based on a single microwave resonator with machine learning. *IEEE Trans. Microw. Theory Techn* **73**(7), 3939–3952 (2025).

[70] Rayas-Sánchez, J. E. et al. Microwave modeling and design optimization: the legacy of John bandler. *IEEE Trans. Microw. Theory Techn*. **73** (1), 87–101 (2025).

[71] Fang, X., Li, H. & Cao, Q. Design of reconfigurable periodic structures based on machine learning. *IEEE Trans. Microw. Theory Techn*. **71** (8), 3341–3351 (2023).

[72] Zhou, Z. et al. A high-quality data acquisition method for machine-learning-based design and analysis of electromagnetic structures. *IEEE Trans. Microw. Theory Techn*. **71** (10), 4295–4306 (2023).

[73] Manfredi, P. Probabilistic uncertainty quantification of microwave circuits using Gaussian processes. *IEEE Trans. Microw. Theory Techn*. **71** (6), 2360–2372 (2023).

[74] Zhang, J. et al. Physics-driven machine-learning approach incorporating Temporal coupled mode theory for intelligent design of metasurfaces. *IEEE Trans. Microw. Theory Techn*. **71** (7), 2875–2887 (2023).

[75] Lim, D. K., Yi, K. P., Jung, S. Y., Jung, H. K. & Ro, J. S. Optimal design of an interior permanent magnet synchronous motor by using a new surrogate-assisted multi-objective optimization. *IEEE Trans. Magn.* **51** (11), 8207504 (2015).

[76] Taran, N., Ionel, D. M. & Dorrell, D. G. Two-level surrogate-assisted differential evolution multi-objective optimization of electric machines using 3-D FEA. *IEEE Trans. Magn* **54**(11), 8107605 (2018).

[77] Koziel, S. & Pietrenko-Dabrowska, A. *Performance-driven surrogate modeling of high-frequency structures* (Springer, 2020).

[78] Koziel, S. Low-cost data-driven surrogate modeling of antenna structures by constrained sampling. *IEEE Antennas Wirel. Prop. Lett.* **16**, 461–464 (2017).

[79] Koziel, S. & Pietrenko-Dabrowska, A. Performance-based nested surrogate modeling of antenna input characteristics. *IEEE Trans. Ant Prop.* **67** (5), 2904–2912 (2019).

[80] Pietrenko-Dabrowska, A. & Koziel, S. Antenna modeling using variable-fidelity EM simulations and constrained co-kriging. *IEEE Access.* **8** (1), 91048–91056 (2020).

[81] Koziel, S. Fast simulation-driven antenna design using response-feature surrogates. *Int. J. RF Micr CAE*. **25** (5), 394–402 (2015).

[82] Koziel, S. & Pietrenko-Dabrowska, A. Expedited feature-based quasi-global optimization of multi-band antennas with Jacobian variability tracking. *IEEE Access.* **8**, 83907–83915 (2020).

[83] Koziel, S. & Bandler, J. W. Reliable microwave modeling by means of variable-fidelity response features. *IEEE Trans. Microw. Theory Tech.* **63** (12), 4247–4254 (2015).

[84] Ullah, U., Koziel, S. & Mabrouk, I. B. Rapid re-design and bandwidth/size trade-offs for compact wideband circular polarization antennas using inverse surrogates and fast EM-based parameter tuning. *IEEE Trans. Ant Prop.* **68** (1), 81–89 (2019).

[85] Marler, R. T. & Arora, J. S. The weighted sum method for multi-objective optimization: new insights. *Struct. Multidisc Opt.* **41**, 853–862 (2010).

[86] Mirjalili, S. & Dong, J. S. *Multi-Objective Optimization using Artificial Intelligence Techniques* (Springer Briefs in Applied Sciences and Technology, 2019).

[87] Mandal, J. K. et al. (eds) *Multi-Objective Optimization: Evolutionary to Hybrid Framework* (Springer, 2018).

[88] Koziel, S. & Pietrenko-Dabrowska, A. Recent advances in accelerated multi-objective design of high-frequency structures using knowledge-based constrained modeling approach. *Knowledge Based Systems* **214**, 106726 (2021).

[89] Cervantes-González, J. C. et al. Space mapping optimization of handset antennas considering EM effects of mobile phone components and human body. *Int. J. RF Microw. CAE*. **26** (2), 121–128 (2016).

[90] Koziel, S. & Ogurtsov, S. *Antenna design by simulation-driven optimization. Surrogate-based approach* (Springer, 2014).

[91] Koziel, S. & Bandler, J. W. A space-mapping approach to microwave device modeling exploiting fuzzy systems .*IEEE Trans. Microwave Theory and Tech 55*(12), 2539–2547 (2007).

[92] Koziel, S., Bandler, J. W. & Madsen, K. Space-mapping based interpolation for engineering optimization. *IEEE Trans. Microw. Theory Tech.54*(6), 2410–2421 (2006).

[93] Koziel, S. & Ogurtsov, S. Model management for cost-efficient surrogate-based optimization of antennas using variable-fidelity electromagnetic simulations. *IET Microwaves Ant Prop.* **6** (15), 1643–1650 (2012).

[94] Pietrenko-Dabrowska, A. & Koziel, S. Accelerated gradient-based optimization of antenna structures using multi-fidelity simulation models. *IEEE Trans. Ant Propag.* **69** (12), 8778–8789 (2021).

[95] Easum, J. A., Nagar, J., Werner, P. L. & Werner, D. H. Efficient multi-objective antenna optimization with tolerance analysis through the use of surrogate models. *IEEE Trans. Ant Propag.* **66** (12), 6706–6715 (2018).

[96] Koziel, S. & Pietrenko-Dabrowska, A. Rapid multi-objective optimization of antennas using nested kriging surrogates and single-fidelity EM simulation models. *Eng. Comp.* **37** (4), 1491–1512 (2019).

[97] Koziel, S. & Pietrenko-Dabrowska, A. Constrained multi-objective optimization of compact microwave circuits by design triangulation and Pareto front interpolation. *European J. Op Research* **299**(1), 302–312 (2021).

[98] Lv, Z., Wang, L., Han, Z., Zhao, J. & Wang, W. Surrogate-assisted particle swarm optimization algorithm with Pareto active learning for expensive multi-objective optimization. *IEEE J. Automatica Sinica*. **6** (3), 838–849 (2019).

[99] Pietrenko-Dabrowska, A. & Koziel, S. „Generalized formulation of response features for reliable optimization of antenna structures. *IEEE Trans. Ant Propag.* **70** (5), 3733–3748 (2021).

[100] Koziel, S. & Pietrenko, A. Rapid design centering of multi-band antennas using knowledge-based inverse models and response features. *Knowledge Based Systems 252*, 109360 (2022).

[101] Conn, A. R., Gould, N. I. M. & Toint, P. L. *Trust Region Methods* (MPS-SIAM Series on Optimization, 2000).

[102] Pietrenko-Dabrowska, A. & Koziel, S. Fast EM-driven parameter tuning of microwave circuits with sparse sensitivity updates via principal directions. *Knowledge-Based Systems* **252**, 109388 (2022).

[103] Levy, H. & Lessman, F. *Finite Difference Equations* (Dover Publications Inc., 1992).

[104] Koziel, S. & Pietrenko-Dabrowska, A. Variable-fidelity simulation models and sparse gradient updates for cost-efficient optimization of compact antenna input characteristics. *Sensors* **19**(8), 1806 (2019). 30991769 10.3390/s19081806 PMC6515375

[105] Broyden, C. G. A class of methods for solving nonlinear simultaneous equations. *Math. Comp.* **19**, 577–593 (1965).

[106] Kennedy, J. & Eberhart, R. C. *Swarm Intelligence* (Morgan Kaufmann, 2001).

[107] Koziel, S. & Pietrenko-Dabrowska, A. Reduced-cost surrogate modeling of compact microwave components by two-level kriging interpolation. *Eng. Opt.* **52** (6), 960–972 (2019).

[108] Phani Kumar, K. V. & Karthikeyan, S. S. „A novel design of rat-race coupler using defected microstrip structure and folding technique, *IEEE Applied Electromagnetics Conf. (AEMC)*, Bhubaneswar, India, pp. 1–2, (2013).

[109] Lin, Z. & Chu, Q. X. „A novel approach to the design of dual-band power divider with variable power dividing ratio based on coupled-lines. *Prog Electromagn. Res.* **103**, 271–284 (2010).

